# Globin gene expression in correlation with G protein-related genes during erythroid differentiation

**DOI:** 10.1186/1471-2164-14-116

**Published:** 2013-02-20

**Authors:** Vladan P Čokić, Reginald D Smith, Angélique Biancotto, Constance T Noguchi, Raj K Puri, Alan N Schechter

**Affiliations:** 1 Laboratory of Experimental Hematology, Institute for Medical Research, University of Belgrade, Dr. Subotica 4, 11129, Belgrade, Serbia; 2 Biosciences Technologies, GE Global Research Center, Niskayuna, NY, USA; 3 Center for Human Immunology, Autoimmunity and Inflammation, National Heart, Lung and Blood Institute, National Institutes of Health, Bethesda, MD, USA; 4 Molecular Medicine Branch, National Institute of Diabetes and Digestive and Kidney Diseases, National Institutes of Health, Bethesda, MD, USA; 5 Division of Cellular and Gene Therapies, Center for Biologics Evaluation and Research, Food and Drug Administration, Bethesda, MD, USA

**Keywords:** G protein, G protein-coupled receptors, Erythroid progenitors, Ontogeny, Globins

## Abstract

**Background:**

The guanine nucleotide binding protein (G protein)-coupled receptors (GPCRs) regulate cell growth, proliferation and differentiation. G proteins are also implicated in erythroid differentiation, and some of them are expressed principally in hematopoietic cells. GPCRs-linked NO/cGMP and p38 MAPK signaling pathways already demonstrated potency for globin gene stimulation. By analyzing erythroid progenitors, derived from hematopoietic cells through in vitro ontogeny, our study intends to determine early markers and signaling pathways of globin gene regulation and their relation to GPCR expression.

**Results:**

Human hematopoietic CD34^+^ progenitors are isolated from fetal liver (FL), cord blood (CB), adult bone marrow (BM), peripheral blood (PB) and G-CSF stimulated mobilized PB (mPB), and then differentiated in vitro into erythroid progenitors. We find that growth capacity is most abundant in FL- and CB-derived erythroid cells. The erythroid progenitor cells are sorted as 100% CD71^+^, but we did not find statistical significance in the variations of CD34, CD36 and GlyA antigens and that confirms similarity in maturation of studied ontogenic periods. During ontogeny, beta-globin gene expression reaches maximum levels in cells of adult blood origin (176 fmol/μg), while gamma-globin gene expression is consistently up-regulated in CB-derived cells (60 fmol/μg). During gamma-globin induction by hydroxycarbamide, we identify stimulated GPCRs (*PTGDR, PTGER1*) and GPCRs-coupled genes known to be activated via the cAMP/PKA (*ADIPOQ*), MAPK pathway (*JUN*) and NO/cGMP (*PRPF18*) signaling pathways. During ontogeny, *GPR45* and *ARRDC1* genes have the most prominent expression in FL-derived erythroid progenitor cells, *GNL3* and *GRP65* genes in CB-derived cells (high gamma-globin gene expression), *GPR110* and *GNG10* in BM-derived cells, *GPR89C* and *GPR172A* in PB-derived cells, and *GPR44* and *GNAQ* genes in mPB-derived cells (high beta-globin gene expression).

**Conclusions:**

These results demonstrate the concomitant activity of GPCR-coupled genes and related signaling pathways during erythropoietic stimulation of globin genes. In accordance with previous reports, the stimulation of GPCRs supports the postulated connection between cAMP/PKA and NO/cGMP pathways in activation of γ-globin expression, via JUN and p38 MAPK signaling.

## Background

The guanine nucleotide binding protein (G protein)-coupled receptor (GPCRs) family represents the largest group of cell surface receptors that regulate cell growth, proliferation, and differentiation [1]. The silencing of *Gpr48*, as GPCR is highly expressed in the fetal liver (FL) and premature erythroblast, has no effects on primitive erythropoiesis but significantly reduces definitive erythropoiesis through the cAMP/PKA/CREB pathway [2]. Inactivation of *Gpr48* induces remarkable decreases in the proliferation of definitive erythroid progenitors and erythroblast islands in FL [2]. GPCRs are linked via G proteins to adenylyl cyclase, phospholipases, and ionic conductance channels [3]. Thus, the Gαs protein is known to couple GPCRs to adenylyl cyclase to stimulate formation of the second messenger cAMP. It has been found that, upon activation of the cAMP pathway, expression of the gamma (γ)-globin gene is induced in adult erythroblasts [4]. Once formed, cAMP consecutively stimulates cAMP-dependent protein kinase (PKA). According to our previous results, cytostatic hydroxycarbamide (hydroxyurea) also induces phosphorylation of endothelial nitric oxide synthase (eNOS) in a PKA-dependent manner [5]. Hydroxycarbamide, as a γ-globin inducer, increases intracellular cAMP levels as well as cGMP levels in human erythroid progenitor cells [6]. Fetal hemoglobin induction by hydroxycarbamide is mediated by the nitric oxide (NO)-dependent activation of soluble guanylyl cyclase (sGC) [7].

G proteins also couple the receptors to other cellular effectors systems. Thus, Gα_o_ has been shown to link GPCRs to Ca^2+^ conductance channels to regulate the influx of Ca^2+^ to cells [8]. Hydroxycarbamide-induced rise in intracellular Ca^2+^ demonstrates dependence on the calcium leak from endoplasmic reticulum [5]. In addition to G proteins, GPCRs also couple with β-arrestins involved in termination of receptor activation after prolonged agonist binding [9]. Furthermore, β-arrestins facilitate the internalization of GPCRs, followed by ubiquitination and proteasome degradation with consequential GPCR down-regulation [10]. We showed that hydroxycarbamide inhibited the proteasome activity, which also supports the correlation between GPCRs and globin genes control [11].

Several groups have examined the gene expression profile of human CD34^+^ hematopoietic progenitor cells from bone marrow (BM), peripheral blood (PB) and cord blood (CB) using microarray technology [12,13]. The modulation of gene expression during ontogeny in FL- and CB-derived hematopoietic progenitor cells appears to overlap largely with early response genes of growth factor stimulated adult BM hematopoietic progenitor cells [14]. Recent studies have begun to define general gene expression profiling of human erythroid cells from different origins - adult BM and PB [15,16]. In general, it has been hypothesized that globin gene switching may be mediated by proteins expressed during different stages of ontogeny.

A previous report demonstrated that stromal feeder layers of human FL, CB, and adult BM did not change hemoglobin types during erythroid differentiation of CD34^+^ hematopoietic progenitor cells derived from the equivalent tissues [17]. Instead of this approach, we perform erythroid differentiation of only CD34^+^ hematopoietic progenitor cells originated from fetal to adult hematopoietic cells. The erythroid cells growth and differentiation markers have been determined in *in vitro* liquid cultures. The γ-globin gene expression is the most increased in CB-derived erythroid cells, while beta (β)-globin gene expression is the highest in adult blood cells (BM, PB) during erythroid differentiation. We compare the G proteins and GPCRs gene expression at several stages in ontogeny by array analyses. During γ-globin induction, we identify GPCRs related genes that were activated via the cAMP/PKA, p38 MAP kinase and NO/cGMP signaling pathways.

## Results

### Characterization of erythroid cell cultures

**Figure 1:**
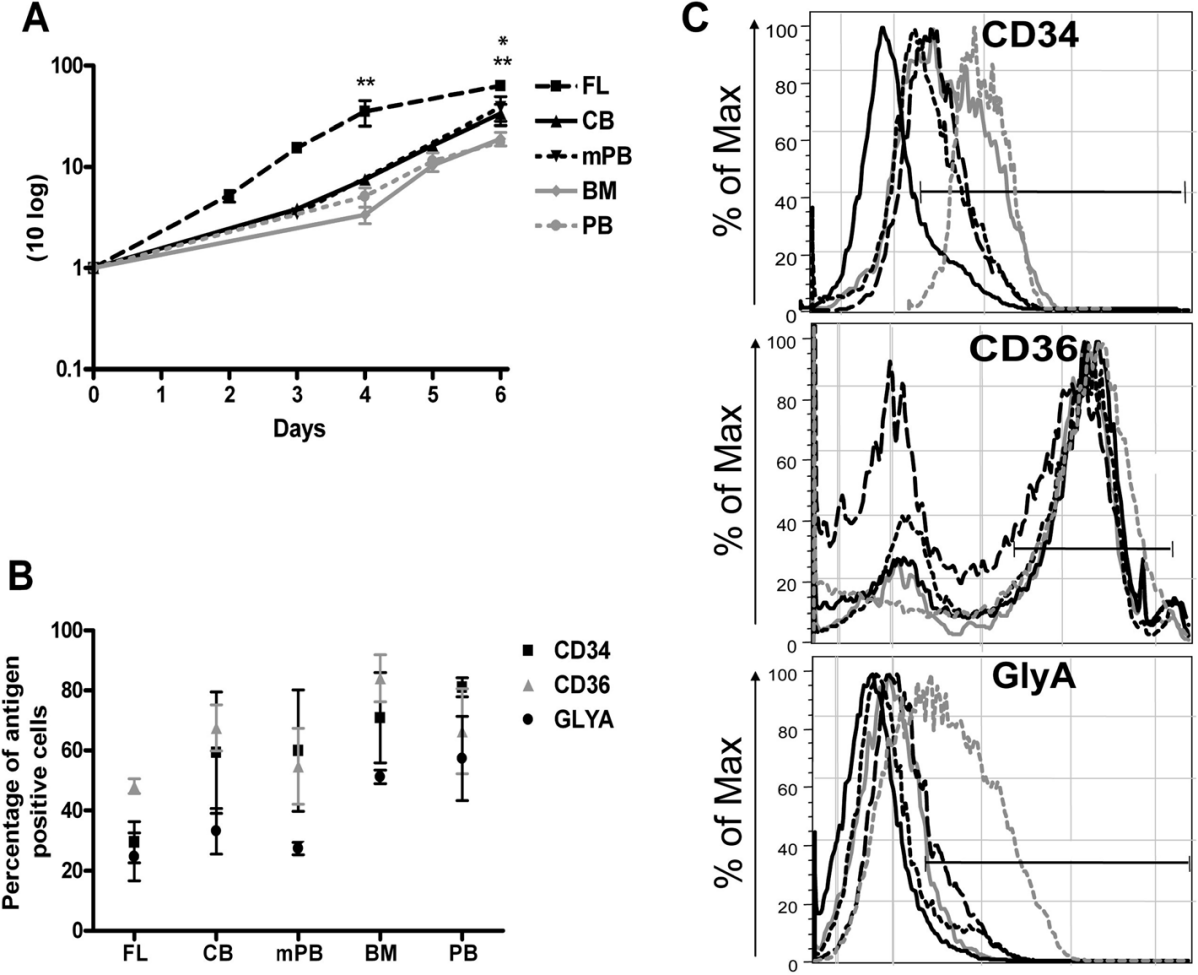
**Cell growth and differentiation of erythroid cells through ontogeny.** (**A**) During erythropoiesis, the cell growth capacity was compared with number of seeded cells in the beginning of culture as fold induction of cell growth on a logarithmic scale. *P < 0.05 FL- versus mPB- and **P < 0.01 FL-derived erythroid cells versus all other cells. (**B**) Flow cytometry of erythroid progenitor cells, sorted as 100% CD71^+^ cells (gated on live CD71^+^ cells), is determined at day 6 of erythroid cultures. Values are mean ± SEM (n=2-4). (**C**) Histograms of CD34, CD36 and GLYA antigen expression on CD71^+^ sorted cells in a representative experiment at day 6 of erythroid cell culture. Values are positive cells for each marker expressed in percentage (%) of maximal number of cells.

Growth potential of CD34^+^ hematopoietic progenitor cells is determined by counting the viable cells. Growth capacity is found to be most abundant for FL-derived CD34^+^ cells during erythroid differentiation. CB- and mPB-derived CD34^+^ cells have a higher cell growth potential than BM- and PB-derived cells during early erythroid differentiation (Figure 1A). In the presence of EPO and other cytokines, CD34^+^ cells are differentiated in vitro into erythroid progenitor cells, confirmed by flow cytometry analysis using four different markers: CD34, CD36, CD71 and GlyA (Figure 1B,C). The transferrin receptor (CD71) is present at early erythroid cells and disappears as reticulocytes differentiate into mature erythrocytes [7]. At day 6 of erythroid cell culture, the erythroid progenitor cells are sorted as 100% CD71^+^, a well-known early marker of erythroid differentiation. Erythroid progenitor cells from all examined cells, except from FL-derived, retain high levels of CD34 antigen expression (about 50–60%). Moreover, all of them and particularly erythroid cells of CB and BM origin, express CD36 antigen (50–70%) also present at early erythroid cells (Figure 1B). We did not find statistical significance in the percentage of CD34, CD36 and GlyA antigen positive cells among erythroid progenitors derived from examined hematopoietic cells (Figure 1B). Besides flow cytometry for analysis of *in vitro* erythroid differentiation, we already reported measurement of hemoglobin content by benzidine staining and high-performance liquid chromatography in erythroid progenitor cells during their *in vitro* differentiation in the same culture conditions [6,7].

### Globin genes expression during erythroid differentiation

**Figure 2:**
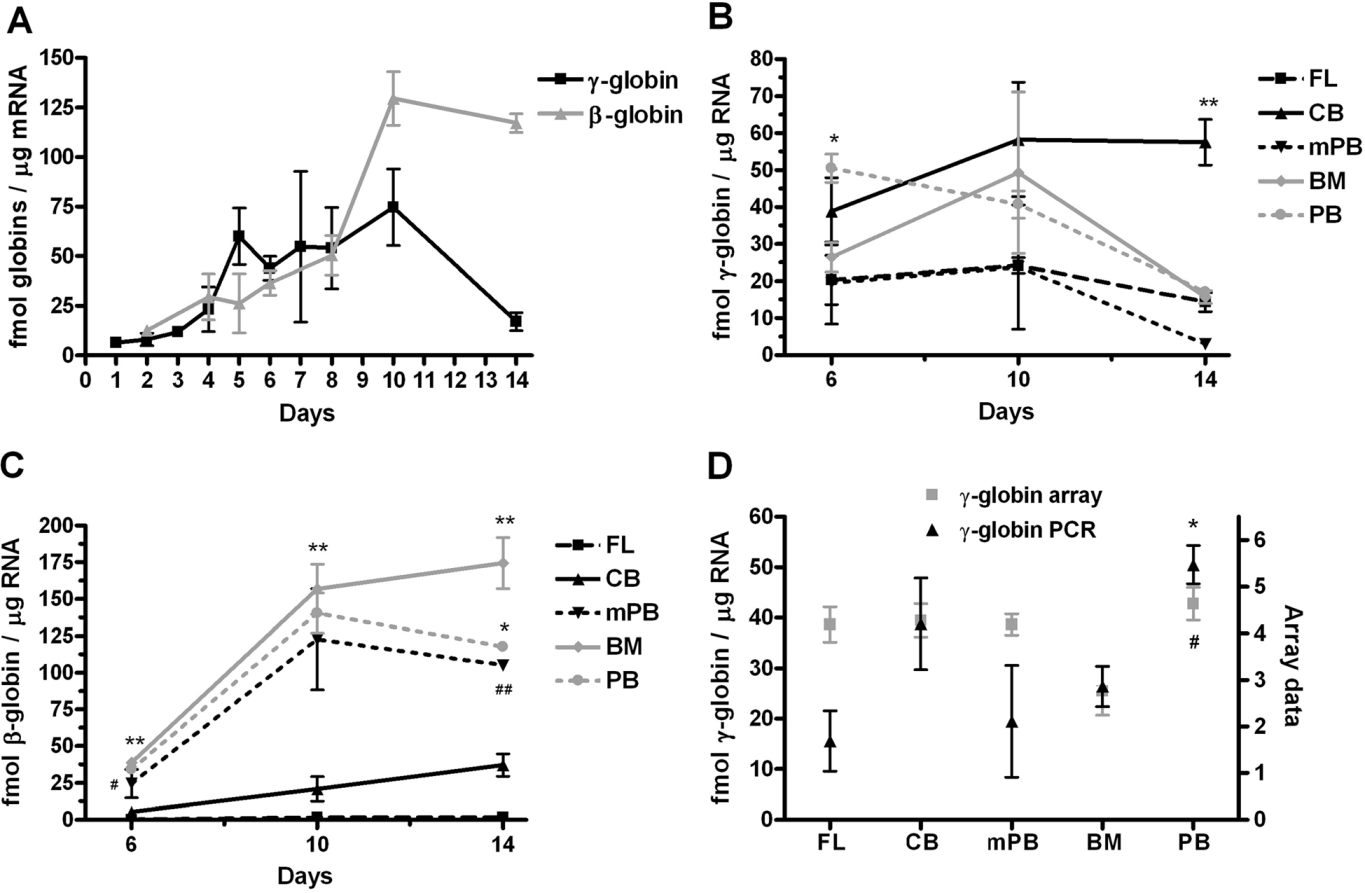
**Measurement of globin genes expression during human erythroid differentiation.** (**A**) Expression of γ- and β-globin genes during erythroid differentiation of CD34^+^ cells derived from normal human PB (n=10). (**B**) Expression of γ-globin gene during erythroid differentiation of hematopoietic CD34^+^ progenitors of FL, CB, BM, PB and mPB origin (n=2-4). *P < 0.05 PB vs FL at day 6, **P < 0.01 CB vs. all other tissues at day 14. (**C**) Expression of β-globin gene during erythroid differentiation (n=2-4). **P < 0.01 FL-, CB-derived erythroid cells vs. all other cells at day 6, 10 and 14, except ^#^P < 0.05 CB vs. mPB at day 6; *P < 0.05 PB vs. BM at day 14; ^##^P < 0.01 mPB vs. BM. (**D**) Comparative expression of γ-globin genes of erythroid progenitor cells as measured by real time quantitative PCR and microarray analysis at day 6 of liquid culture (n=2-4). ). *P < 0.05 PB vs. FL by qPCR, ^#^P < 0.05 BM vs. PB by microarray. Values are mean ± SEM.

In erythroid progenitor cells derived from PB, γ- and β-globin gene expression is initially similar, but during erythroid maturation β-globin gene expression becomes elevated in erythroid precursor cells and mature erythroid cells (Figure 2A). We already described erythroid differentiation stages, using erythroid specific cell surface markers [7]. The γ-globin gene expression in erythroid cells, of FL origin, has stable expression during erythroid differentiation (about 20 fmol/μg), whereas erythroid cells of CB demonstrate elevation (about 60 fmol/μg). The γ-globin gene expression of adult hematopoietic progenitor-derived erythroid cells demonstrates reduction during differentiation reaching minimum at day 14 (3–15 fmol/μg), while its expression was at maximum in CB-derived erythroid cells (Figure 2B). The β-globin gene expression is almost absent in erythroid progenitors of FL origin, whereas its expression is slightly increased in erythroid cells of CB origin (35 fmol/μg at day 14) and reaches maximum in cells of adult blood origin: BM (176 fmol/μg) and PB (110 fmol/μg) (Figure 2C). To compare quantitative PCR results and microarray data of γ-globin gene expression, we combine them to demonstrate their expected similar tendencies during ontogeny (Figure 2D). At day 6 of erythroid culture, the γ-globin gene expression is the most elevated in CB- and PB-derived erythroid progenitors, as determined by quantitative PCR. The lowest level of γ-globin gene expression is in BM-derived erythroid progenitors, as determined by microarray (Figure 2D).

### Effect of hydroxycarbamide on G protein-coupled receptor signaling pathways in human erythroid progenitor cells

**Figure 3:**
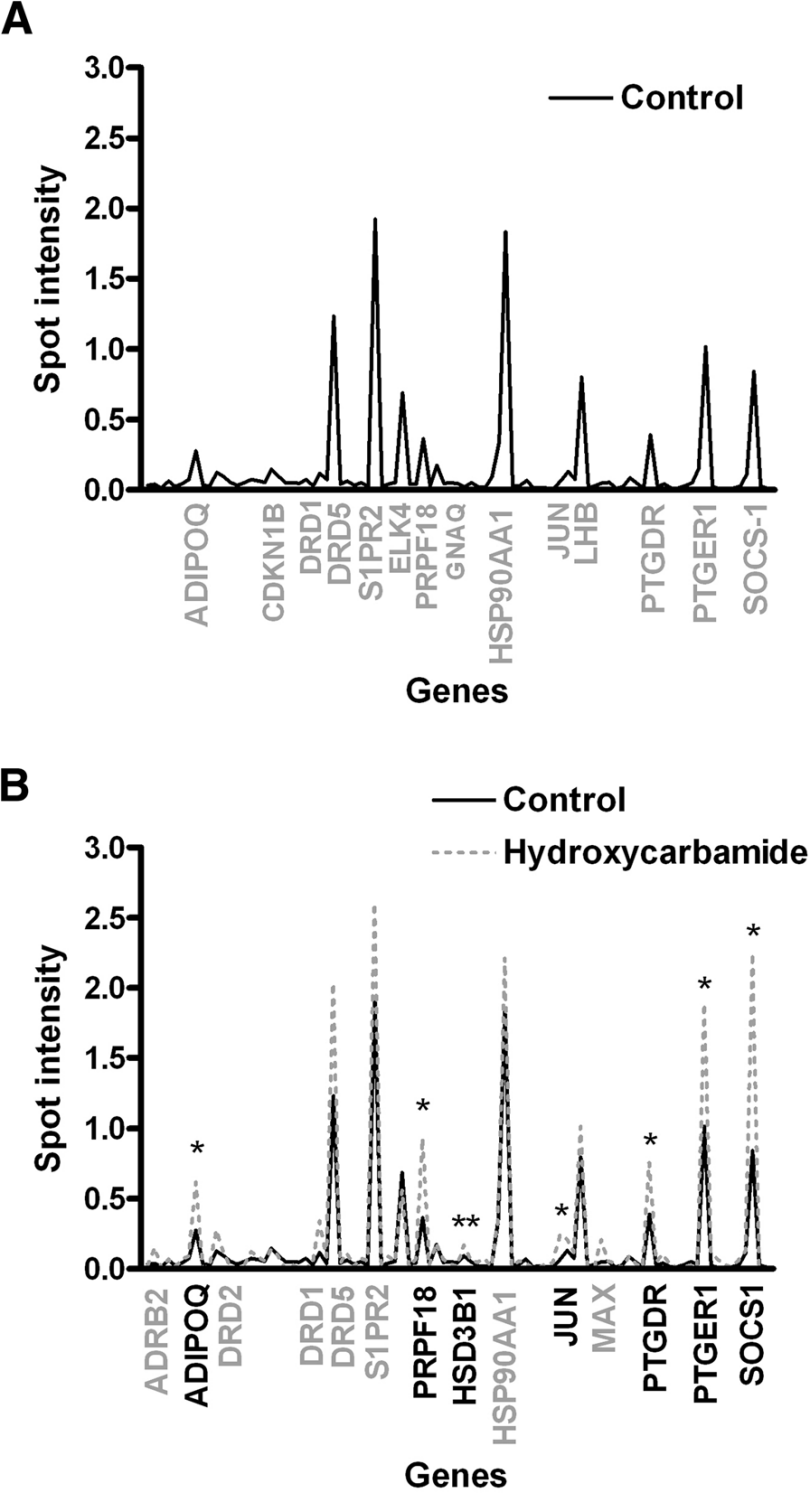
**Human G protein-coupled receptors signaling pathway profile in erythroid progenitor cells.** (**A**) Human GPCRs Signaling Pathway related genes with increased expression at day 6 of erythroid progenitor cell culture of PB origin. Gene expression was normalized to cyclophilin A. Control represents untreated cells. (**B**) We analyzed erythroid progenitors at day 6 of culture after 48 hours of treatment with 40 μM hydroxycarbamide. We presented only hydroxycarbamide-induced genes using their symbols. Results are from three independent experiments. *P < 0.05. Values are mean ± SEM.

It has been reported that hydroxycarbamide, a γ-globin inducer, activated p38 mitogen-activated protein kinase (MAPK) and c-jun expression [18]. Since these pathways are also activated by GPCRs, we are interested to determine additional related pathways in PB-derived erythroid progenitor cells in a steady state and after incubation with hydroxycarbamide using GPCRs Signaling Pathway Finder Gene Array at day 6 of erythroid culture (Table 1). In the steady state, we find elevated gene expression of *DRD5, S1PR2, PTGDR* and *PTGER1* in human erythroid progenitor cells (Figure 3A). We also find increase in gene expression activated by the cAMP/PKA pathway (*ADIPOQ*), PKC pathway (*LHB* and *ELK4*), MAPK pathway (*YWHAZ*), NO-cGMP pathway (*PRPF18*) and JAK-STAT pathway (*HSPA4, SOCS1* and *HSP90AA1*). *HSP90AA1* has the steady upregulated levels during ontogeny in erythroid cells, as well as *HSPA4* and *YWHAZ* gene expression (not shown). Hydroxycarbamide treatment of erythroid progenitor cells induces the statistically significant expression of following GPCRs: *PTGDR* (1.9 fold), and *PTGER1* genes (1.9 fold, Figure 3B). Hydroxycarbamide also stimulates gene expression activated by cAMP/PKA pathway (*ADIPOQ*, 3.1 fold), NO/cGMP pathway (*PRPF18*, 2.6 fold), MAPK pathway (*JUN*, 1.6 fold) and JAK-STAT pathway (*SOCS1*, 2.7 fold, Figure 3B). Hydroxycarbamide increases γ-globin gene expression up to 2.5 fold in erythroid progenitor cells used for GPCRs Gene Array, as measured by real-time quantitative PCR [7]. Gene expression of IL8, upregulated by hydroxycarbamide, is also elevated in erythroid cells during ontogeny and more prominent in BM tissue, as determined by microarray analysis (not shown).

**Table 1:** Human G protein-coupled receptor signaling pathway profile in erythroid progenitor cells, of peripheral blood origin, after treatment with hydroxycarbamide

**UniGene**	**Symbol**	**Description**	**Gene expression**		
**G protein-coupled receptors:**			**Control±SD**	**HU±SD**	**p**
Hs.2551	ADRB2	Beta 2 adrenergic receptor	0,04±0,04	0,15±0,1	0.107
Hs.2624	DRD1	D1 dopamine receptor	0,12±0,1	0,34±0,2	0.1206
Hs.73893	DRD2	D2 dopamine receptor	0,13±0,02	0,28±0,1	0.0715
Hs.380681	DRD5	D5 dopamine receptor	1,23±0,4	2,03±1,1	0.2115
Hs.458474	S1PR2	Sphingosine-1-phosphate receptor 2	1,92±0,4	2,6±1,1	0.2614
Hs.306831	PTGDR	Human DP prostanoid receptor	0,39±0,1	0,76±0,1	**0.0391**
Hs.159360	PTGER1	Prostaglandin E receptor 1, EP1 subtype	1,02±0,2	1,89±0,5	**0.0305**
**PI-3 kinase pathway:**					
Hs.525622	AKT1	v-akt murine thymoma viral oncogene homolog 1	0,04±0,01	0,07±0,06	0.139
**cAMP/PKA pathway:**					
Hs.80485	ADIPOQ	Adiponectin, C1Q and collagen domain containing	0,28±0,09	0,87±0,2	**0.0432**
**NO/cGMP pathway:**					
Hs.161181	PRPF18	PRP18 pre-mRNA processing factor 18 homolog	0,36±0,28	0,93±0,6	**0.0497**
**PKC pathway (Ca**^**2+**^**,MEK, etc.):**					
Hs.154704	LHB	Luteinizing hormone beta polypeptide	0,8±0,1	1±0,4	0.1705
Hs.25292	JUNB	Jun B proto-oncogene,	0,08±0,08	0,12±0,1	0.0915
Hs.624	IL8	Interleukin 8	0,07±0,04	0,24±0,3	0.1875
Hs.497520	ELK4	ETS-domain protein (SRF accessory protein 1)	0,69±0,12	0,56±0,2	0.1441
**JAK-STAT pathway:**					
Hs.90093	HSPA4	Heat shock 70 kDa protein 4	0,34±0,1	0,35±0,14	0.4318
Hs.525600	HSP90AA1	Heat shock 90 kDa protein 1, alpha	1,84±0,5	2,21±1,2	0.3635
Hs.50640	SOCS1	Suppressor of cytokine signaling 1	0,84±0,4	2,25±0,96	**0.0341**
Hs.364941	HSD3B1	Hydroxy-delta-5-steroid dehydrogenase	0,09±0,02	0,17±0,03	**0.005**
**MAP kinase pathway (p42/p44MAP, p38MAP):**					
Hs.414795	SERPINE1	Plasminogen activator inhibitor, type I	0,11±0,1	0,13±0,06	0.3309
Hs.525704	JUN	v-jun avian sarcoma virus 17 oncogene homolog	0,13±0,04	0,21±0,01	**0.0193**
Hs.492407	YWHAZ	Tyrosine 3-monooxygenase/tryptophan 5-monooxygenase activation protein	1,36±0,3	1,28±0,3	0.3155
Hs.285354	MAX	Human helix-loop-helix zipper protein	0,05±0,01	0,21±0,2	0.1637

HU – hydroxycarbamide, p – P value of paired t test of Control vs. HU.

### G-protein related genes in erythroid progenitor cells during ontogenesis

**Figure 4:**
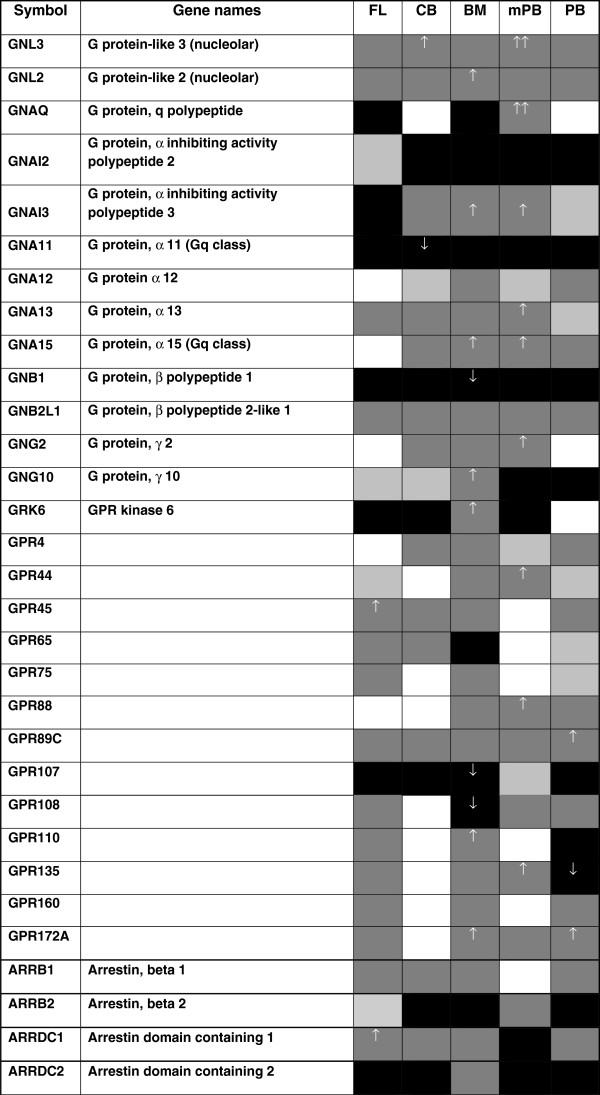
**G proteins and GPCRs expression in erythropoiesis during ontogenesis.** Black boxes – decreased gene expression versus HuURNA, dark gray boxes – increased gene expression, light gray boxes – no change in gene expression, white boxes – absent gene expression. G protein-coupled receptor (GPR), guanine nucleotide binding protein (G protein), up arrow - significantly increased and down arrow - decreased gene expression among examined erythroid progenitors.

The G protein superfamily consists of heterotrimeric complexes of distinct α-, β-, and γ-subunits. Heterotrimeric G proteins are classified according to α subunit into four subfamilies: Gs, Gi, Gq, and G_12/13_[3]. To distinguish significant G proteins and GPCRs during erythropoiesis we performed microarray analysis of erythroid progenitor cells in certain stages of human ontogeny. During microarray analysis the genes are upregulated or downregulated versus reference HuURNA, what we use as a control alongside each sample. G-protein related genes: *GNAI2* and *GNB1* are continuously downregulated vs. *GNB2L1* upregulation during ontogeny (Figure 4). *GNAI3* gene expression was prominently upregulated in BM- and mPB-derived cells, but downregulated in FL-derived cells. Steady upregulation also demonstrates *GNL2* and *GNL3* genes. The increased *GNG10* and *GRK6* gene expression is observed in BM-derived cells (Figure 4), with elevated β-globin gene expression (Figure 2C). The expression of *GNAQ* gene is significantly increased only in erythroid progenitors of mPB origin. In addition, GPCRs genes: *GPR65* gene has decreased gene expression only in BM-, whereas *GPR135* only in PB-derived cells. *GPR45* gene has elevated expression in erythroid cells of FL origin, whereas *GPR108* has decreased expression in BM-derived cells, opposite to *GPR110* and *GPR172A* genes. In addition, β-arrestins are involved in termination of GPCRs activation after prolonged agonist binding [9]. *ARRDC1* gene expression is the most upregulated in FL- and downregulated only in mPB-derived cells, whereas *ARRDC2* expression is decreased in all examined erythroid progenitors except of BM origin (Figure 4). The microarray data discussed in this publication we deposited in NCBI’s GEO database and are accessible through GEO Series accession number GSE37869 (http://www.ncbi.nlm.nih.gov/geo/query/acc.cgi?acc=GSE37869).

## Discussion

To reveal the essential mechanisms in erythropoiesis several studies have performed gene expression profiling in erythroid cells from hematopoietic tissues through ontogeny. The findings in these reports are heterogeneous, reflecting the variation in the experimental systems used. By choosing early erythroid progenitors differentiated from purified CD34^+^ cells, we extend those studies to evaluate globin gene expression in correlation to GPCR-coupled genes from fetal to adult erythropoiesis. The γ-globin gene expression is most prominent in CB-derived erythroid progenitors, whereas β-globin gene expression is major in adult blood-derived (BM, PB, mPB), low in CB-derived and almost completely absent in FL-derived erythroid progenitors. The GPCR-coupled genes have been studied in erythroid progenitor cells during stimulation of γ-globin gene production. Hydroxycarbamide stimulates the expression of several genes (*ADIPOQ, SOCS1, HSP90AA1, PRPF18*) activated by GPCRs via cAMP/PKA, p38 MAPK, and NO/cGMP signaling pathways. A certain number of G-proteins (α, β, γ isoforms) and GPCRs demonstrate variation or stability of gene expression in erythroid progenitor cells during ontogeny. Genes *GPR45* and *ARRDC1* have the most prominent expression in FL-derived, genes *GNL3* and *GRP65* in CB-derived (with high γ-globin gene expression), *GNL2, GPR110, ARRDC2* and *GNG10* in BM-derived (with β-globin gene expression), *GNAQ, GNA13* and *GPR44* in mPB-derived, *GPR89C* and *GPR172A* in PB-derived erythroid progenitors.

**Figure 5:**
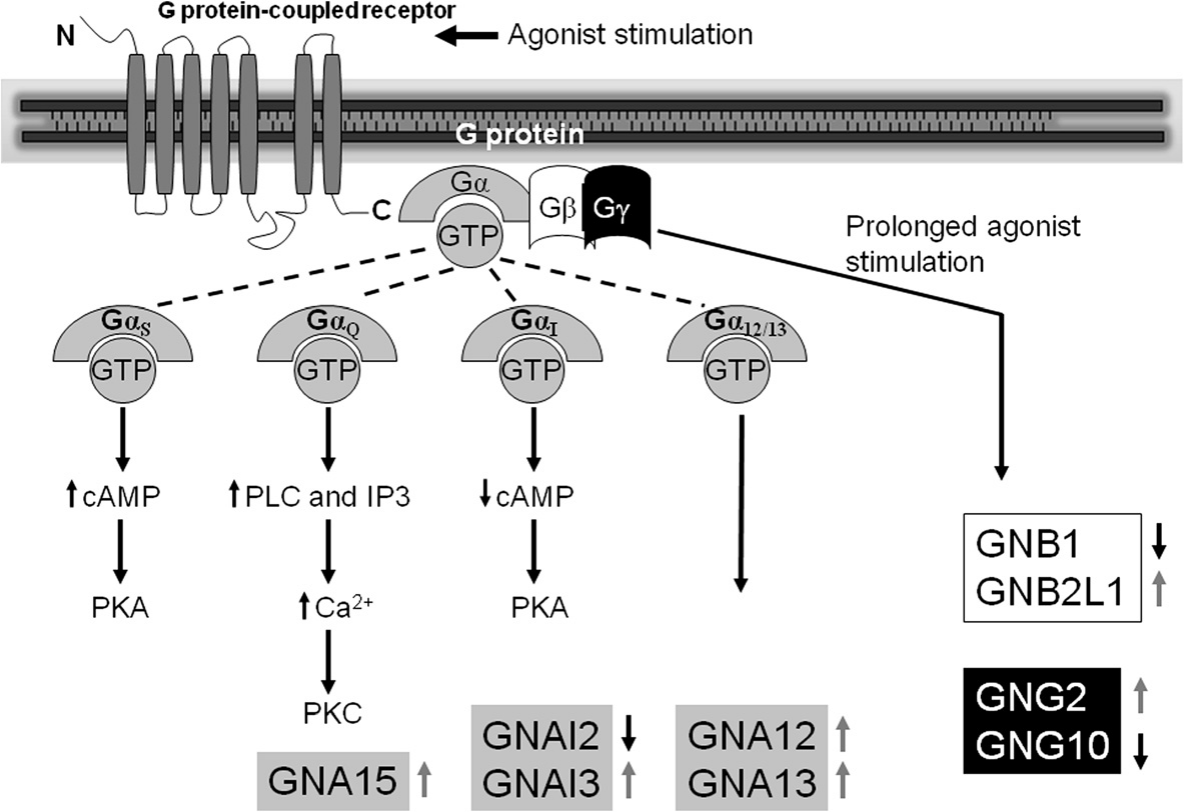
**Principle of GPCR signaling.** Acute agonist stimulation of GPCRs leads to activation of multiple signaling pathways, including second messengers cAMP, IP3 and Ca^2+^. GPCRs in the plasma membrane couple to these pathways via G proteins that connect the receptors to enzymes adenylyl cyclase, phospholipase C (PLC) or ionic conductance channels such as Ca^2+^ channels. Prolonged stimulation of the receptor leads to recruitment to the cell membrane of *β*-adrenergic receptor kinases and *β*-arrestin. Detected genes by microarray are presented in colored boxes corresponding to G protein isoforms (α, β, γ). The detected genes are upregulated (gray arrow) or downregulated (black arrow) vs. HuURNA through ontogeny. PKA, protein kinase A; PKC, protein kinase C.

As previously reported, G protein expression and MAPKs are involved in hemin-induced erythroid differentiation, another γ-globin stimulator [19]. Hydroxycarbamide increased phosphorylation of p38 in erythroid differentiation [18]. In accordance with hydroxycarbamide and G protein linked activity, Gα_12/13_ also induced activation of p38 MAPK (Figure 5) [20]. It has been revealed that the p38 activation resulted in the stimulation of NF-kappaB-specific DNA-protein binding and the subsequent expression of inducible NOS and NO release [21]. Moreover, G protein α_12_ (*GNA12*) stimulation of the eNOS protein expression is in accordance with hydroxycarbamide induction of eNOS protein levels (Figure 5) [11,22]. We have previously shown that fetal hemoglobin stimulation, by hydroxycarbamide, is dependent on NO/cGMP signaling pathway in erythroid progenitor cells [7]. In addition, the activated G protein α_13_ (*GNA13*) induced the NF-kappaB accompanied by augmented secretion of IL-8 (Figure 5) [23]. Induced expression of *IL-8, HSPA4, SERPINE1* and *JUN* by hydroxycarbamide, achieved in our experiments, is also observed upon hemin-induced erythroid differentiation [24]. It has been reported that hydroxycarbamide increased *JUN* gene and protein expression in erythroid cells, by increasing the rate of synthesis as well as stabilizing the mRNA [25]. Initial EPO-dependent *JunB* induction was not sufficient, but the late EPO-independent *JunB* expression was necessary for differentiation of primary erythroid cells [26]. *GNA13* gene expression has been elevated in erythropoiesis throughout ontogeny, while *GNA12* gene expression is prominent in erythroid progenitors of adult cells origin (Figure 4).

The G-protein α_15_ (*GNA15*) is expressed particularly in hematopoietic cells [27]. Beta 2-adrenergic receptor (*ADRB2*), induced by hydroxycarbamide, can specifically couple to *GNA15* upregulated in erythroid progenitors of CB and adult cells origin in our microarray study [28]. It has been reported that calcium-sensing receptor-mediated MAP kinase (ERK1/2) activation requires *GNAI2* coupling [29]. Inhibition of the ERK pathway lead to increased hemoglobin levels [30]. Furthermore, according to our results *GNAI2* gene expression is decreased in every examined erythroid progenitors. The activation of p38 MAPK signaling pathway, induced by *GNA12/13*, is also involved in butyrate-mediated erythroid differentiation, another γ-globin inducer as well as inhibitor of histone deacetylases [31]. Additionally, the inhibition of histone deacetylases induced a high increase of γ-globin mRNA and activated p38 signaling during fetal hemoglobin stimulation [32].

**Figure 6:**
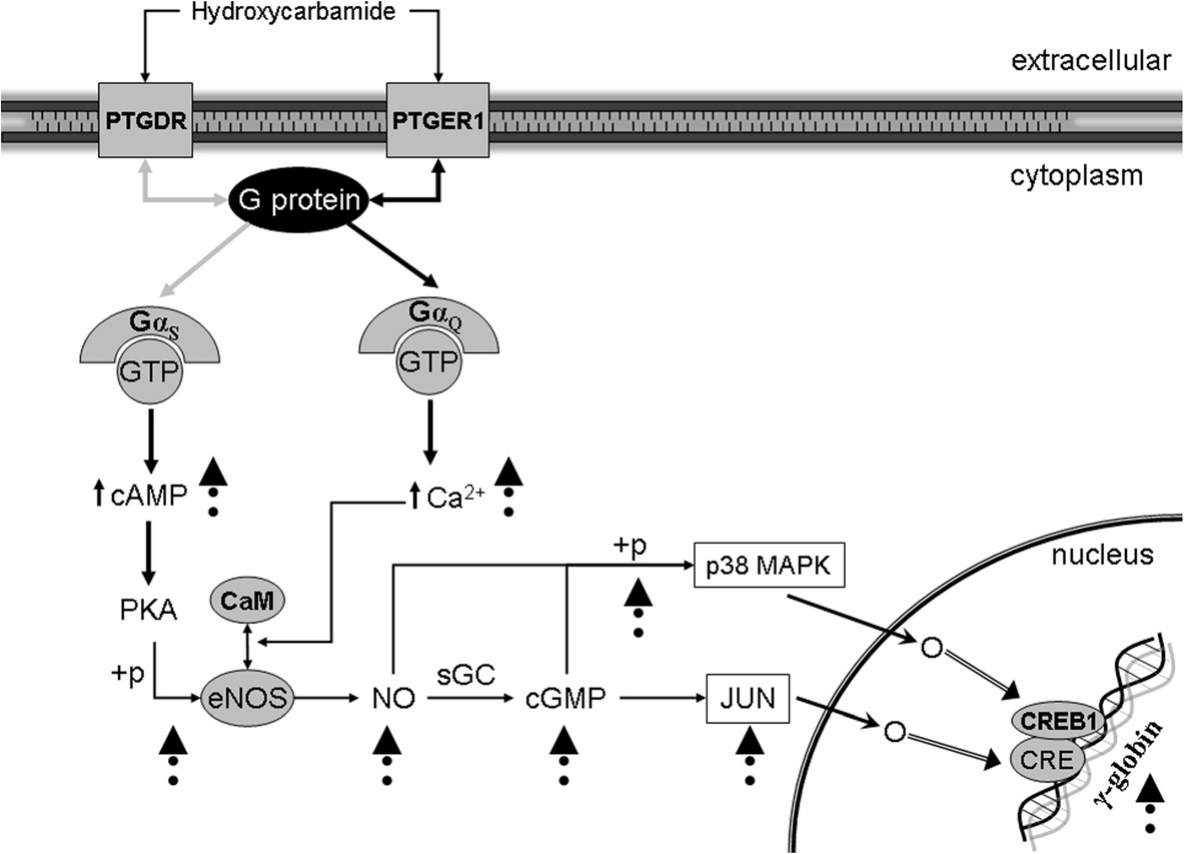
**Induction of gamma-globin gene expression via GPCR signaling and NO-cGMP pathway.** Stimulation of GPCRs (*PTGDR* and *PTGER1*) in the plasma membrane leads to activation of NO-cGMP pathway via G proteins that connect the receptors to enzymes adenylyl cyclase or ionic conductance channels generating second messengers’ cAMP and Ca^2+^. PKA – cAMP dependent protein kinase; CaM – calmodulin; CRE - cAMP response element; (+p) phosphorylation, → stimulation, →O - translocation, ▲- experimentally documented hydroxycarbamide induction.

We show that hydroxycarbamide stimulates GPCRs *PTGDR* and *PTGER1* gene expression. The activity of *PTGDR* receptor is mediated by Gα_S_ proteins that stimulate adenylate cyclase resulting in an elevation of intracellular cAMP and Ca^2+^, while *PTGER1* mediates activity through Gα_Q_ proteins that stimulate phosphatidylinositol-calcium second messenger system (Figure 6) [33,34]. It has been reported that expression of the γ-globin gene is induced upon activation of the cAMP pathway, while adenylate cyclase inhibition markedly decreased fetal hemoglobin induction by hydroxycarbamide in human erythroid cells [4,35]. Hydroxycarbamide increased intracellular cGMP as well as cAMP levels in human erythroid progenitors, while cAMP consecutively stimulates PKA [11]. Phosphorylation of eNOS at Ser1177 by hydroxycarbamide was completely PKA dependent, accompanied by a rise of the intracellular Ca^2+^ concentration [5]. Besides phosphorylation, eNOS production of NO is also stimulated by increased intracellular Ca^2+^ levels and interaction with calmodulin (CaM). We demonstrated that NO increased γ-globin gene expression in human erythroid cells during differentiation. In addition, inhibition of sGC prevents NO and hydroxycarbamide stimulation of γ-globin gene expression [7]. Furthermore, sGC activators or cGMP analogs augmented γ-globin gene expression in primary human erythroblasts [36]. The NO–cGMP pathway is known to increase *JUN* mRNA levels [37]. We show that hydroxycarbamide increases GPCR-coupled *JUN* gene expression, which is in accordance with previous studies related to induction of *JUN* and γ-globin gene expression (Figure 6) [25,38]. It has been also postulated that *JUN* activates the Gγ-globin promoter via an upstream cAMP response element (CRE) in a way comparable to transcription factor CRE binding protein 1 (*CREB1*) [39]. *CREB2*, a key transcription factor in erythropoiesis, was down-regulated in Gpr48−/− fetal livers through the cAMP-PKA-CREB pathway, with decreased adult hemoglobin α and β chains [2]. There have been previous reports of p38 MAPK increased phosphorylation by both NO and cGMP [40,41]. Hydroxycarbamide also augmented phosphorylation of p38, and demonstrated dependence of p38 activity during stimulation of fetal hemoglobin production [18]. Finally, the *CREB1* activated γ-globin expression via p38 MAPK signaling in erythroid progenitors [42,43]. This observation emphasizes the mutual stimulation of GPCRs and NO/cGMP pathway, via *JUN* and p38 MAPK signaling, in activation of γ-globin expression (Figure 6).

## Conclusions

While mechanisms involved in globin gene expression have been recognized at different levels within the regulatory hierarchy, relations among these molecular pathways are only emerging. We associate our new results with the NO/cGMP pathway described in our previous publications [6,7], and demonstrate the induction of G-proteins and GPCR-coupled genes during γ-globin stimulation and erythropoiesis through ontogeny. These genes and related signaling pathways, involved in the mechanism of globin activation, might be targets for the therapeutic agents to upregulate γ-globin gene expression and fetal hemoglobin levels in hemoglobinopathies. Therefore, further direct studies are required to confirm that modifications in the level of expression of GPCRs lead to significant changes in NO/cGMP and other signaling pathways important for γ-globin gene expression in erythroid cells.

## Methods

### Liquid erythroid cell cultures

For erythroid progenitor cell cultures, blood was obtained from consenting normal volunteers from the National Institutes of Health, Department of Transfusion Medicine according to the regulations and guidelines of the Office of Human Subjects Research. Adult PB mononuclear cells are isolated from buffy coats of healthy donors (3 individuals per experiment) using Lymphocyte Separation Medium (BioWhittaker, Walkersville, MD). We wash mononuclear cells twice with Dulbecco’s phosphate-buffered saline (PBS, Invitrogen Corporation, Carlsbad, CA), and CD34^+^ hematopoietic progenitors are purified by positive immunomagnetic selection using the MACS cell isolation system (Miltenyi Biotec, Auburn, CA). Commercial FL- (Cambrex Bio Science, Inc., Walkersville, MD), CB-, BM- and granulocyte-colony stimulating factor (G-CSF) stimulated mobilized PB- (mPB, AllCells LLC, Berkeley, CA) derived CD34^+^ cells are also collected by positive immunomagnetic selection (Miltenyi Biotec). To stimulate erythroid differentiation, the labeled CD34^+^ cells of all samples are cultured in the medium that contains 30% fetal bovine serum (FBS), 2 mmol/L glutamine, 100 U/ml penicillin, 100 μg/ml streptomycin, 10% deionized bovine serum albumin, 10 mmol/L mercaptoethanol, 1 mmol/L dexamethasone, 33 μg/ml holo-transferrin, 10 ng/ml SCF, 1 ng/ml IL-3 and 1 ng/ml GM-CSF (Sigma, St. Louis, MO), and 1 U/ml human recombinant EPO (Amgen Inc, Thousand Oaks, CA) [7]. For microarray analysis, erythroid progenitors are isolated at day 6 of erythroid cell culture at 37°C and 5% CO_2_ with balanced 95% room air. At different time points during *in vitro* erythroid differentiation, the viable cell counts are performed with the use of a trypan-blue exclusion technique (BioWhittaker).

### Immunofluorescence analysis

After 6 days of erythroid culture, 5×10^5^ cells are washed in PBS containing 0.5% FCS and 0.02% sodium azide and incubated for 20 minutes at the ambient temperature in the presence of the appropriate monoclonal antibodies at a twofold saturating concentration. Anti-glycophorin-A (GlyA) FITC, anti-CD34 PE, anti-CD71 Tricolor, anti-CD36-APC markers are used for cell staining at day 6 of erythroid culture (Beckman-Coulter, Miami, FL). Erythroid cells are then washed, fixed in PBS containing 4% formaldehyde, and acquired on an LSRII flow cytometer (BD Biosciences, San Jose, CA) equipped with lasers emitting at wavelengths 355, 488, 532, 407 and 638 nm using DIVA4.1.2 software. The saturation of unspecific binding sites is achieved by normal mouse serum control present in the staining buffer. Data are analyzed with Flowjo software (Tree Star, San Carlos, CA).

### Isolation of total RNA

After 6 days of erythropoietin treatment and incubation at 37°C (5% CO_2_, 95% humidity), we use the RNeasy protocol for isolation of total RNA from erythroid progenitor cells (Qiagen, Valencia, CA) according to the manufacturer’s instructions. Concentration and integrity of total RNA is assessed using an 8453 UV/Visible Spectrophotometer (Hewlett-Packard GmbH, Waldbronn, Germany) and Agilent 2100 Bioanalyzer Software (Agilent Technologies, Waldbronn, Germany) comparing the ratio of 28S and 18S RNA peaks to ensure that there is minimal degradation of the RNA sample. One microgram of total RNA is reverse-transcribed with SuperScript II RNase H^-^ Reverse Transcriptase (Invitrogen Corporation).

### Quantitative PCR

Quantitative real-time PCR assay of γ- and β-globin mRNA transcripts is carried out with the use of gene-specific double fluorescently labeled probes in a 7700 Sequence Detector (Applied Biosystems, Foster City, CA). The specific primers and TAQMAN probes (synthesized by the NIDDK core oligonucleotide facility) are designed using Primer Express software (Applied Biosystems) and prepared on an ABI 394 synthesizer (Applied Biosystems) as previously described [44]. Platinum Quantitative PCR SuperMix-UDG (Invitrogen Corporation) is used for each of the primer pairs containing a final concentration of 200 μM dNTPs, 0.5 μM Rox reference dye (Invitrogen Corporation), 0.2 μM each of TAQMAN probe, forward and reverse primers. Expression levels are determined using the associated SDS software (ABI Prism, Applied Biosystems) and Microsoft Excel (Redmond, WA). Standard curves are constructed using dilutions of an accurately determined plasmid containing the cDNA of interest as template.

### Microarray studies

In microarray studies, the numbers of total genes overexpressed in erythroid cells of CB, BM and PB origin are determined from three independent samples as biological repeats at day 6 of erythroid liquid culture. On the other hand in case of FL and mPB-derived samples, the numbers of total overexpressed genes are determined in independent duplicate samples at day 6 of erythroid liquid culture. High quality oligonucleotide glass arrays are produced containing a total of 16,659 seventy-mer oligonucleotides chosen from 750 bases of the 3^′^ end of each ORF (Operon Inc. Valencia, CA). The arrays are produced in house by spotting oligonucleotides on poly-L-lysine coated glass slides by Gene Machines robotics (Omnigrid, San Carlos, CA). We have followed the MIAME (minimum information about a microarray experiment) guidelines for the presentation of our data [45].

i) Probe preparation

Total human universal RNA (HuURNA) isolated from a collection of adult human tissues to represent a broad range of expressed genes from both male and female donors (BD Biosciences, Palo Alto, CA) serve as a universal reference control in the competitive hybridization. All 5 blood tissues are hybridized against HuURNA. The correlation coefficients among those biological repeats themselves are consistently ≥ 0.8, which documented the quality of hybridization and consistency of expression among the replicates of all 5 tissues. Labeled cDNA probes are produced as described [46].

ii) Hybridization

For hybridization, 36 μl hybridization mixture (cDNA mixture, 10 μg COT-1 DNA, 8–10 μg poly(dA), 4 μg l yeast total RNA, 20X SSC and 10% SDS) is pre-heated at 100°C for 2 minutes and cooled for 1 minute. Total volume of probe is added on the array and covered with cover slip. Slides are placed in hybridization chamber and 20 μl water is added to the slide, and incubated overnight at 65°C. Slides are then washed for 2 minutes each in 2X SSC, 1X SSC and 0.1X SSC and spin-dried.

iii) Data Filtration, normalization, and analysis

Microarray slides are scanned in both Cy3 (532 nm) and Cy5 (635 nm) channels using Axon GenePix 4000B scanner (Axon Instruments, Inc., Foster City, CA) with a 10-micron resolution. Scanned microarray images are exported as TIFF files to GenePix Pro 3.0 software for image analysis. For advanced data analysis, gpr and jpeg files are imported into microarray database, and normalized by software tools provided by NIH Center for Information Technology (http://nciarray.nci.nih.gov). We gathered a set of 8,719 erythroid cells gene expression data derived from 11 datasets that have been posted on the National Center for Biotechnology Information (NCBI) Gene Expression Omnibus (GEO) database.

### Human G Protein-coupled receptors signaling pathway finder gene *array*

Erythroid progenitor cells, of PB origin, are treated with hydroxycarbamide at day 4 after stimulation with EPO and incubated 48 hours at 37°C. At day 6, total RNA is isolated. GPCRs array is completed using the GEArray Q Series Chemiluminescence Detection User system (SABiosciences, Frederick, MD). Briefly, the preheated annealing mix with 2 μg of total RNA is added to labeling mix with biotin-16-dUTP (Roche Applied Science, Indianapolis, IN) and reverse transcriptase (Promega Corporation, Madison, WI). The biotin labeled cDNA probe is denatured before addition of the probe to the hybridization solution with the GEArray Q Series membrane. After hybridization with continuous agitation, and washing, the chemiluminescent detection is performed. We use a digital imaging system (FluorChem Imaging system, Alpha Innotech Corporation, San Leandro, CA) to record the chemiluminescent image of the array. The relative abundance of a particular transcript is estimated by directly comparing its signal intensity to the signal derived from a housekeeping gene cyclophilin A. The list of 110 genes for human GPCRs signaling pathway finder gene array is available from the manufacturer (SABiosciences, Frederick, MD).

### Statistical analysis

The one way ANOVA Tukey’s Multiple Comparison tests and paired t test are applied using Prism 4 software (GraphPad Software Inc., San Diego, CA) for measurement of statistical significance in cell growth and antigen levels among blood tissues, as well as for γ- and β-globin expression. For microarray data management and analysis, we use NCI/CIT microArray database (mAdb) system.

## Competing interests

The authors declare that they have no competing interests.

## Authors’ contributions

VPC carried out experimental work described in the paper, participated in designing the study, drafted the manuscript and performed the statistical analysis. RDS carried out the quantitative real-time PCR assays and performed the statistical analysis. AB carried out the immunofluorescence analysis and performed the statistical analysis. CTN participated in designing the study and coordination and helped to draft the manuscript. RKP conceived of the study, and participated in its design and coordination and helped to draft the manuscript. ANS participated in designing the study and the writing of the manuscript. All authors read and approved the final manuscript.
